# The Fabrication of Protein Carriers for Intracellular Delivery of Antibiotics Against Intracellular Bacterial Infection

**DOI:** 10.3390/molecules31132215

**Published:** 2026-06-24

**Authors:** Ting Pan, Baozhu Wang, Haojie Du, Yuhan Yan, Kai Zhang, Cheng Chi, Ronggui Lu, Risheng Li, Yong-Miao Shen, Li Hao, Zhijun Zhang

**Affiliations:** 1 Department of Chemistry, School of Chemistry and Chemical Engineering, Zhejiang Sci-Tech University, Hangzhou 310018, China; 2 Shengzhou Innovation Research Institute of Zhejiang Sci-Tech University, Shengzhou 312400, China; 3 Zhejiang Dongbang Pharmaceutical Co., Ltd., Linhai 317022, China

**Keywords:** miniprotein ZF5.3, bovine serum albumin, intracellular delivery, click chemistry, intracellular bacterial infection

## Abstract

Bacterial infections pose a serious threat to human health, and antibiotics remain the first-line therapeutic agents in clinical practice. However, the vast majority of antibiotics lack the ability to penetrate cell membranes, which severely limits the number of clinically available options for treating intracellular bacterial infections. Developing efficient intracellular antibiotic delivery strategies is therefore of considerable clinical significance, both for reducing antibiotic dosage and for expanding the repertoire of drugs applicable to intracellular infections. To address this challenge, we constructed a protein-based delivery platform mediated by a cell-penetrating miniprotein for efficient intracellular antibiotic delivery. In this system, bovine serum albumin (BSA), which possesses broad antibiotic-binding capability, was employed as the drug carrier, while the cell-penetrating miniprotein ZF5.3, which is capable of endosomal escape, served as the transmembrane delivery mediator. ZF5.3 was conjugated to BSA via a bioorthogonal reaction, and ceftriaxone (CRO) was selected as the model antibiotic to construct a nanoscale delivery system. The binding interaction between CRO and BSA was characterized using UV-Vis, HPLC, and molecular docking techniques. The assembly of the ZF5.3–BSA delivery platform was confirmed by UV-Vis absorption spectroscopy and gel electrophoresis. Intracellular delivery efficiency was evaluated by confocal fluorescence imaging and flow cytometry, and the results demonstrated that ZF5.3 conjugation enhanced intracellular protein delivery efficiency by over 5-fold. Fluorescence co-localization analysis revealed that ZF5.3-mediated cargo is mainly distributed in the cytoplasm and does not completely co-localize with lysosomal markers, suggesting its ability to effectively escape from lysosomes. An intracellular infection model using *Staphylococcus aureus* was established. Colony-forming unit (CFU) counting experiments confirmed that the delivery system significantly enhanced the intracellular antibacterial activity of ceftriaxone. CCK8 cytotoxicity assays confirmed that the system is non-toxic to cells.

## 1. Introduction

Intracellular bacterial infection represents a major clinical challenge in anti-infective therapy [1,2]. Pathogenic bacteria such as *Staphylococcus aureus* are capable of invading host cells and persisting intracellularly, thereby evading host immune clearance and the action of conventional antibiotics [3,4,5]. This leads to recurrent, refractory infections and substantially increases the risk of developing antimicrobial resistance [6]. Antibiotics remain the cornerstone of clinical antibacterial therapy, and over the past several decades, various classes of antibiotics—including cephalosporins and fluoroquinolones [7]—have undergone continuous development and iteration. Modern antibiotics generally exhibit broad-spectrum antibacterial coverage and potent bactericidal activity [8,9,10]. However, with the exception of a few agents such as rifampicin, the vast majority of clinically used antibiotics have poor cell membrane permeability and are unable to reach effective bactericidal concentrations within host cells, rendering them largely ineffective against intracellular pathogens [11]. Moreover, the therapeutic efficacy against intracellular parasitic bacteria is limited. In addition, due to the high risk of recurrent infection caused by intracellular bacteria [12,13,14], long-term continuous administration is required in clinical treatment, which significantly increases the risk of developing drug-resistant strains and may eventually lead to a scenario where no effective drugs are available [15,16]. To address the clinical challenges in treating intracellular bacterial infections, besides developing novel antibiotics with cell-penetrating ability, the development of efficient intracellular delivery systems for antibiotics is also of great clinical significance and offers better timeliness [17].

Intracellular drug delivery systems typically comprise a drug carrier and a cell-penetrating modification (transmembrane mediator) as the two core components [18]. Proteins have been widely adopted as drug carriers owing to their excellent biocompatibility, biodegradability, and abundance of functional modification sites [19]. In addition to surface-exposed reactive groups such as amino and carboxyl moieties, albumins such as BSA possess natural hydrophobic cavities that enable efficient non-covalent loading of small-molecule drugs, facilitating stable drug encapsulation and controlled release [20,21,22]. To date, albumins such as BSA have been extensively employed in the construction of various drug delivery systems, some of which have already entered clinical use, highlighting their promising clinical application prospects [23]. The cell membrane permeability of drug carriers is often conferred by modifying them with highly cationic polymers or peptides [24]. Although effective, the strong electrostatic interactions that facilitate cellular entry of the drug carrier may also disrupt the cell membrane structure, leading to cytotoxicity [25]. Moreover, most polymers and cell-penetrating peptides lack the ability to escape from lysosomes, rendering the drugs prone to degradation and inactivation [26]. Recently, an artificially designed miniprotein, ZF5.3, has demonstrated excellent properties in cell membrane penetration [27]. ZF5.3 is a zinc-finger protein composed of 27 residues [28], which folds into a stable conformation through a unique peptide sequence. Unlike conventional cell-penetrating peptides such as TAT and R9 [29], which primarily rely on endocytic pathways, ZF5.3 efficiently enters the cytoplasm via an allosterically mediated endosomal escape mechanism, thereby circumventing lysosomal degradation of the cargo [30]. Studies have demonstrated that ZF5.3 exhibits significantly superior intracellular delivery capacity compared to TAT and R9, while maintaining low cytotoxicity and high biosafety. These characteristics highlight the strong potential of ZF5.3 as a transmembrane mediator for intracellular drug delivery.

**Scheme 1:**
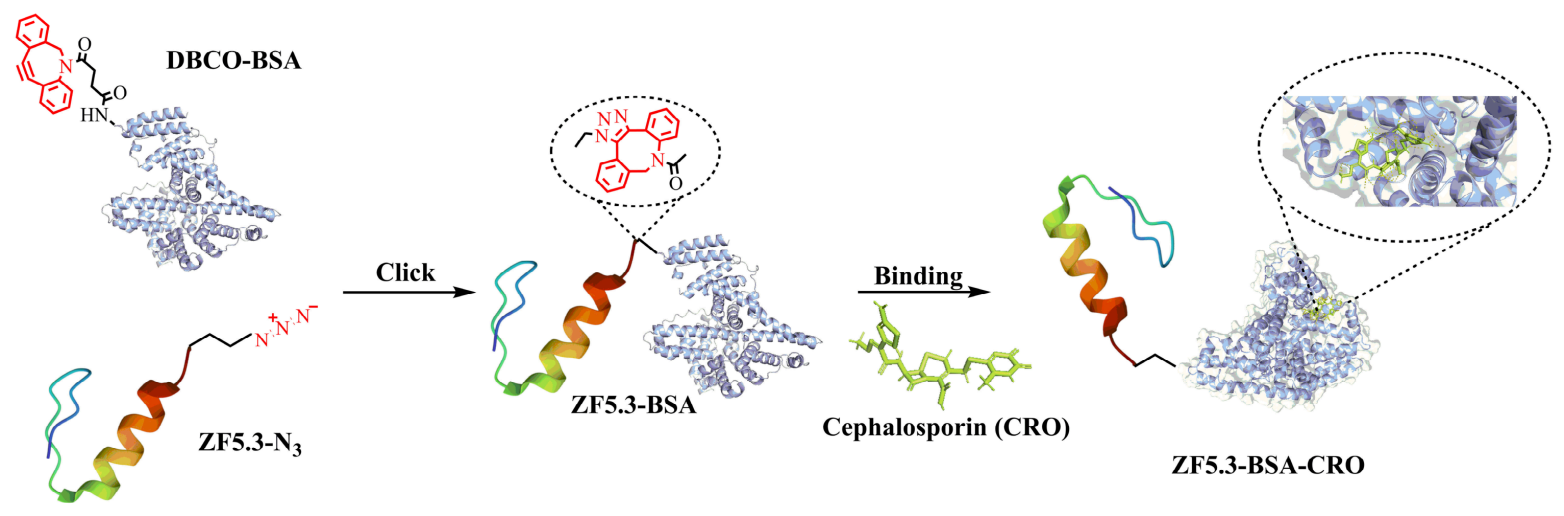
Schematic illustration of the fabrication of protein carrier for intracellular delivery of antibiotics.

Therefore, this study constructed a ZF5.3-mediated protein delivery platform for efficient intracellular delivery of antibiotics. In this system, BSA, which has a broad ability to bind antibiotics, was used as the drug carrier, and the cell-penetrating miniprotein ZF5.3, which possesses lysosomal escape capability, served as the transmembrane delivery mediator. ZF5.3 was bioorthogonally conjugated to BSA via click chemistry, and ceftriaxone, a third-generation cephalosporin antibiotic with potent and broad-spectrum antibacterial activity, was selected as a model antibiotic to construct the delivery system (Scheme 1).

## 2. Results and Discussion

### 2.1. Synthesis and Characterization of ZF5.3

**Figure 1:**
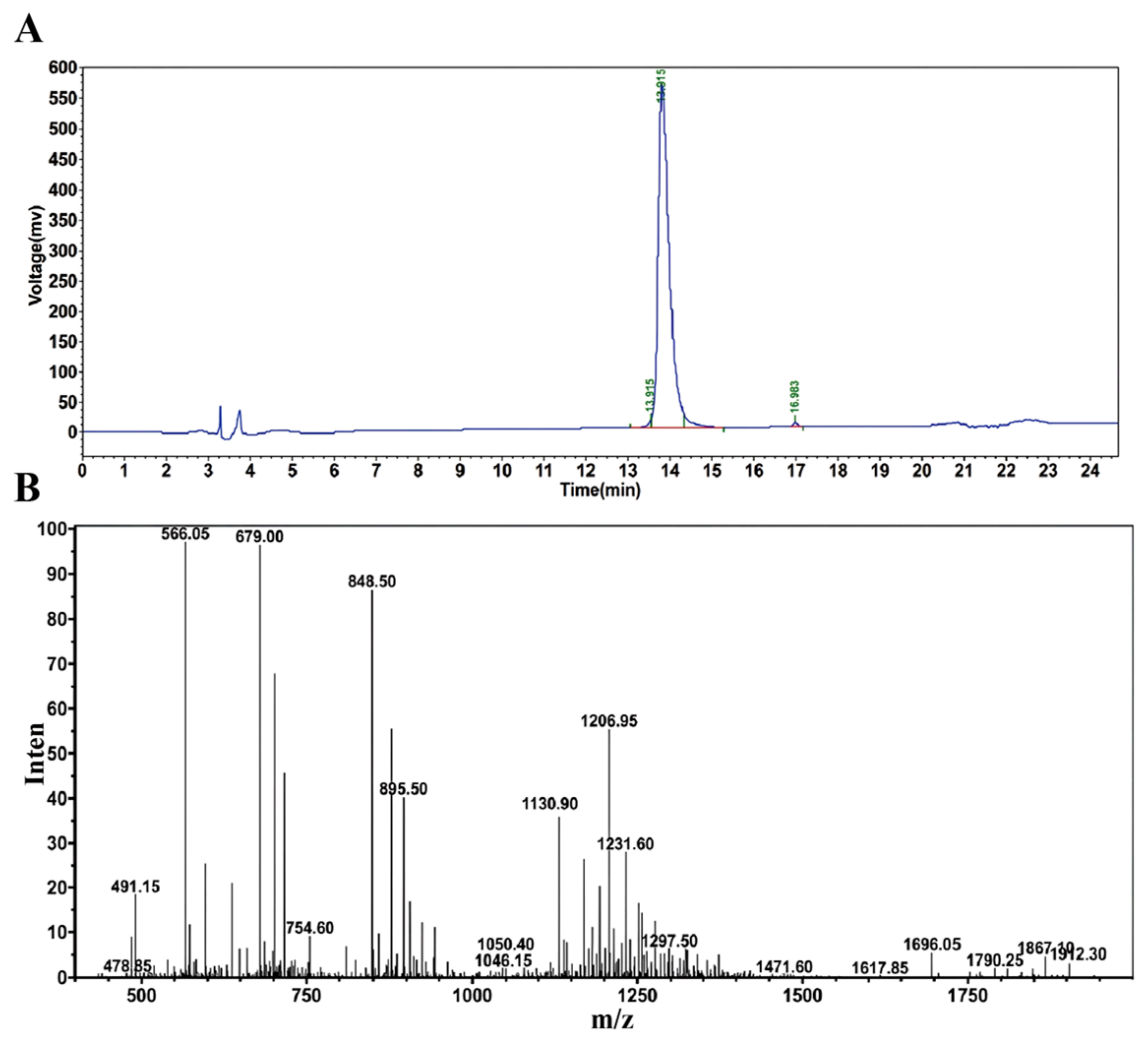
Characterization of ZF5.3. (**A**) High-performance liquid chromatography (HPLC) characterization; (**B**) mass spectrometry (MS) characterization.

ZF5.3 was synthesized by Fmoc solid-phase peptide synthesis. To facilitate bioorthogonal conjugation with BSA, an azide-modified lysine residue was incorporated at the C-terminus during synthesis. The purified ZF5.3 was characterized by analytical HPLC and LC-MS, and the results are shown in Figure 1. The HPLC chromatogram (Figure 1A) revealed a single predominant peak at a retention time of 13.82 min, with a peak area ratio of 95.86%, indicating high purity of the obtained product. In the mass spectrum (Figure 1B), multiple charge state ions were observed at *m*/*z* 1130.90, 848.50, and 679.00, corresponding to [M + 3H]^3+^, [M + 4H]^4+^, and [M + 5H]^5+^, respectively. The calculated molecular weight of 3389.87 Da was in close agreement with the theoretical value of 3389.93 Da, confirming that the synthesized miniprotein was consistent with the designed sequence. Taken together, the HPLC and mass spectrometry results demonstrate the successful preparation of high-purity ZF5.3 suitable for subsequent experiments.

### 2.2. Synthesis and Characterization of the ZF5.3-BSA Conjugate

**Figure 2:**
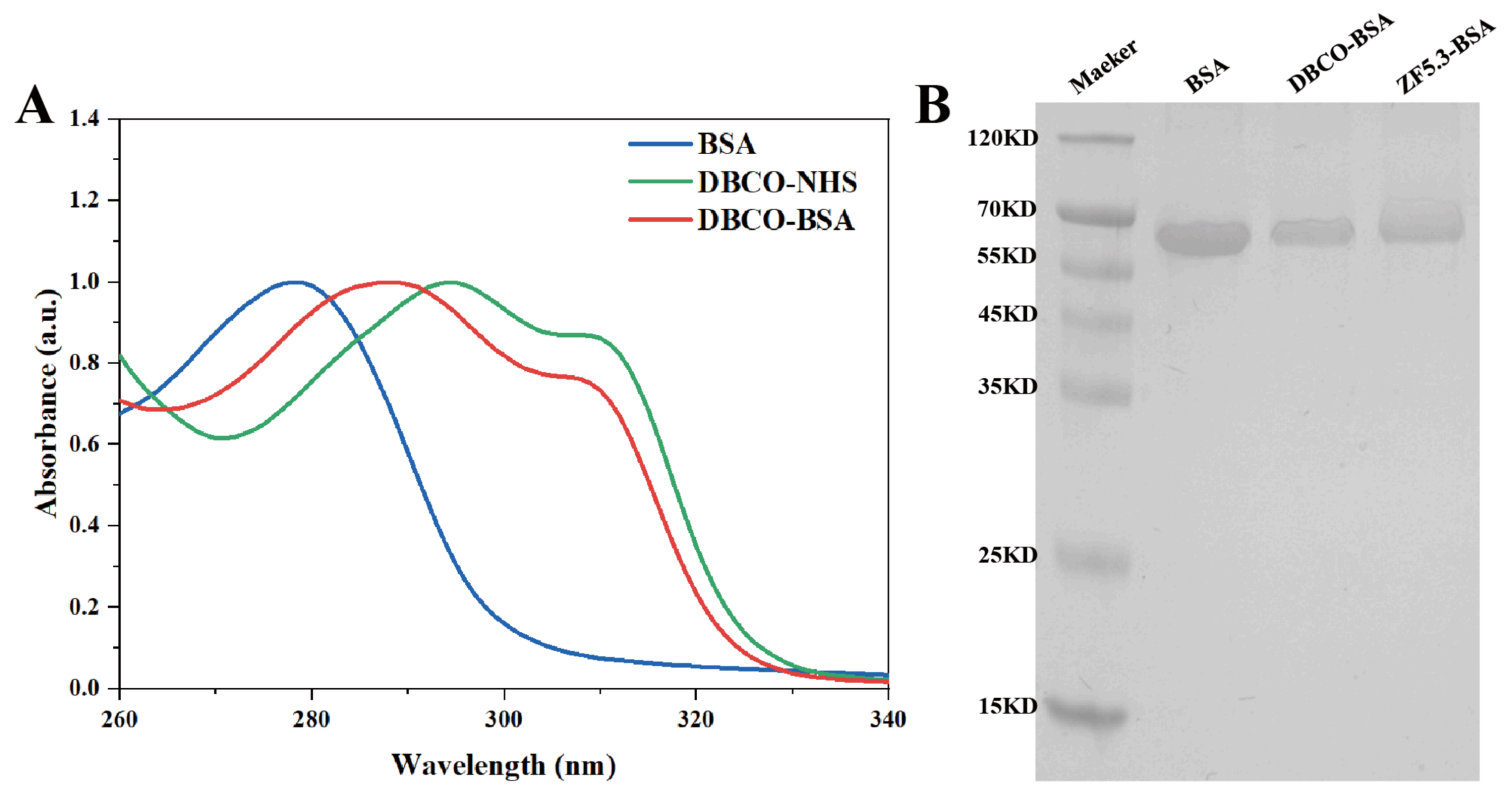
Characterization of ZF5.3-BSA. (**A**) UV-Vis absorption spectra of BSA, DBCO-NHS, and DBCO-BSA; (**B**) SDS-PAGE results of BSA, DBCO-BSA, and ZF5.3-BSA.

Prior to conjugation with ZF5.3, BSA was modified with DBCO groups. DBCO-functionalized BSA (DBCO-BSA) was prepared by reacting BSA with DBCO-NHS ester followed by ultrafiltration purification. The DBCO modification was confirmed by UV-Vis absorption spectroscopy (Figure 2A). Native BSA displayed a characteristic protein absorption peak at 280 nm, while DBCO-NHS exhibited two absorption peaks at 295 and 310 nm. After conjugation, the absorption spectrum of DBCO-BSA showed the characteristic features of both the protein and DBCO moiety, confirming successful preparation of the DBCO-BSA conjugate. ZF5.3 was subsequently conjugated to DBCO-BSA via azide-alkyne click chemistry to obtain ZF5.3-BSA. SDS-PAGE analysis (Figure 2B) showed that native BSA migrated as a main sharp band at approximately 66 kDa. After DBCO modification, the DBCO-BSA band showed a slight upward shift relative to native BSA. After conjugation with ZF5.3, the ZF5.3-BSA conjugate migrated at approximately 70 kDa, indicating that the conjugation reaction between ZF5.3 and DBCO-BSA proceeded efficiently. These results provide a reliable material foundation for subsequent targeted delivery and intracellular antibacterial experiments. Furthermore, the homogeneity of the complexes was further verified by native polyacrylamide gel electrophoresis combined with silver staining. The results showed that both BSA-CRO and ZF5.3-BSA-CRO migrated as single, sharp bands without smearing (Figure S1), indicating that the complexes were intact and homogeneous.

### 2.3. Evaluation of Intracellular Uptake Efficiency of ZF5.3-BSA

**Figure 3:**
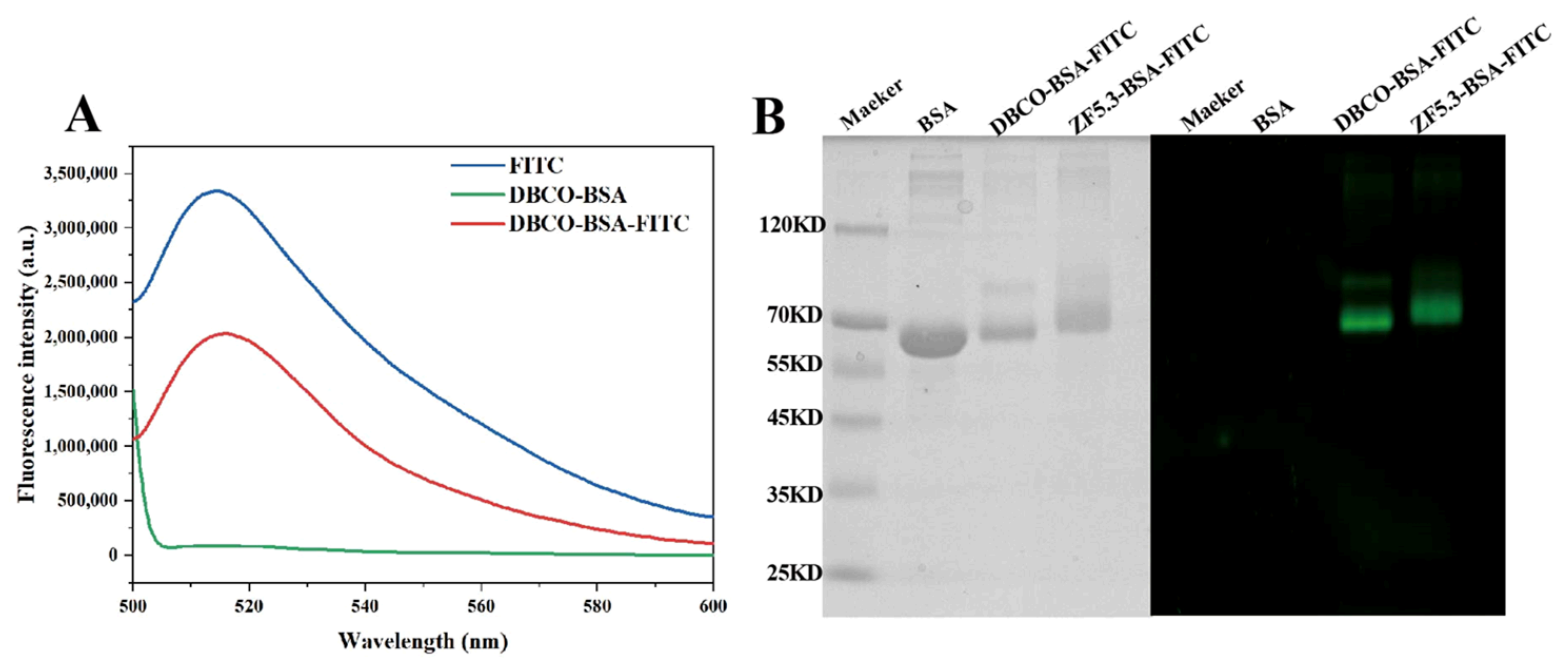
Synthesis of ZF5.3-BSA-FITC conjugate and characterization of FITC labeling. (**A**) Fluorescence emission spectra of FITC, DBCO-BSA and DBCO-BSA-FITC; (**B**) SDS-PAGE and fluorescence images of BSA, DBCO-BSA-FITC, and ZF5.3-BSA-FITC.

To enable fluorescence-based monitoring of cellular uptake, FITC-labeled BSA conjugates were prepared. DBCO-BSA was first labeled with FITC to yield DBCO-BSA-FITC, which was subsequently conjugated with ZF5.3 to produce ZF5.3-BSA-FITC. Successful preparation of the fluorescently labeled conjugates was confirmed by fluorescence emission spectroscopy and gel electrophoresis imaging (Figure 3).

**Figure 4:**
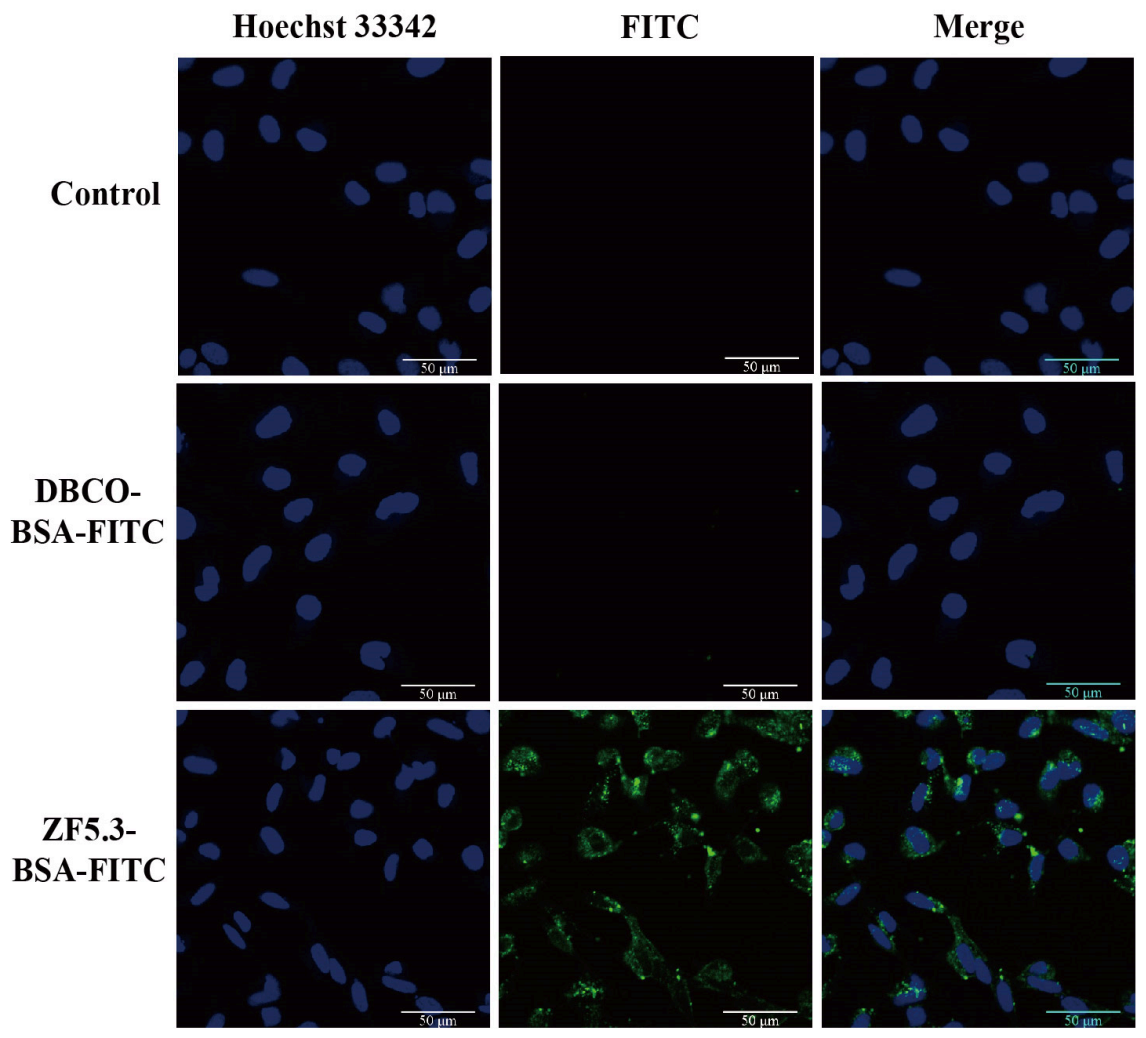
Laser confocal microscopy imaging of HeLa cells in different treatment groups (blue: Hoechst 33342 nuclear staining; green: FITC-labeled protein, scale bar = 50 μm).

Subsequently, we investigated the effect of ZF5.3 modification on cellular uptake using laser scanning confocal microscopy. As shown in Figure 4, no appreciable green fluorescence signal was detected in the untreated control cells. Cells treated with DBCO-BSA-FITC exhibited only faint punctate fluorescence, indicating limited capacity to penetrate the cell membrane. In contrast, cells treated with ZF5.3-BSA-FITC displayed intense and diffuse green fluorescence distributed predominantly throughout the cytoplasm and perinuclear region, directly demonstrating that ZF5.3 significantly enhances the cellular internalization of BSA.

**Figure 5:**
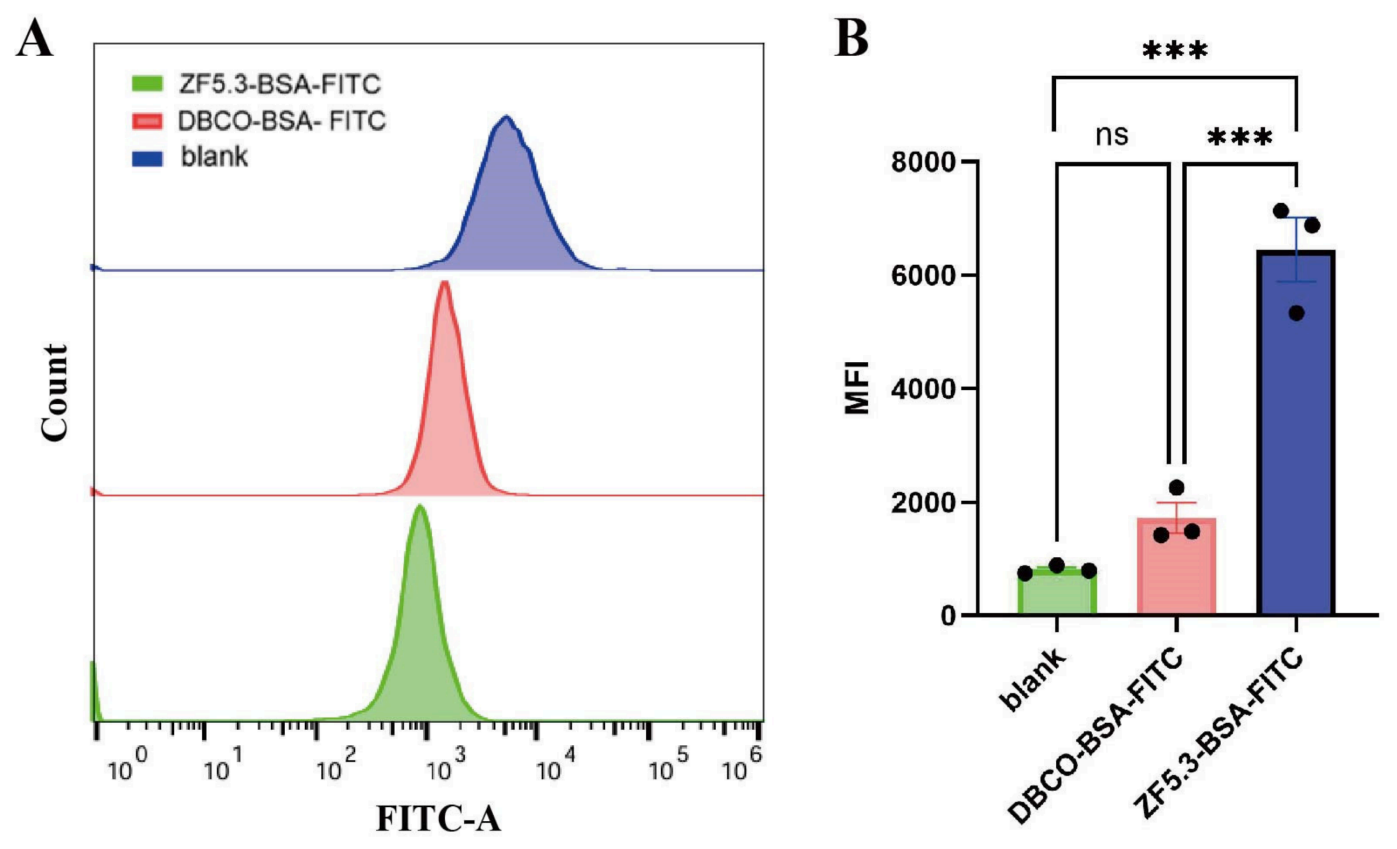
Flow cytometric analysis of intracellular fluorescence. (**A**) Intracellular fluorescence intensity distribution detected by flow cytometry; (**B**) quantitative statistics of mean fluorescence intensity (MFI) in different groups. Data are presented as mean ± standard deviation (SD), n = 3 biological replicates. ns, no significant difference; *** *p* < 0.001.

Flow cytometric analysis was subsequently performed to quantify intracellular fluorescence intensity (Figure 5A,B). The mean fluorescence intensity of cells treated with ZF5.3-BSA-FITC was significantly higher than that of the DBCO-BSA-FITC group, with an enhancement exceeding 5-fold.

Collectively, these results confirm that ZF5.3 possesses outstanding cell-penetrating capability, significantly enhancing the intracellular delivery efficiency of BSA. In HeLa cells, ZF5.3 improved the delivery efficiency by more than 5-fold, far superior to conventional cell-penetrating peptides such as TAT or R9, which typically achieve only 2- to 3-fold enhancement. This superior performance is mainly attributed to the unique allosterically mediated endosomal escape mechanism of ZF5.3, rather than the endocytic pathway that relies solely on electrostatic adsorption. We further verified the enhanced cellular uptake of ZF5.3 in 3T3 cells, and the results were consistent with those obtained in HeLa cells (Figure S2). To directly validate its lysosomal escape capability, we performed a colocalization assay in 3T3 cells. Fluorescence co-localization analysis revealed that ZF5.3-mediated cargo is mainly distributed in the cytoplasm and does not completely co-localize with lysosomal markers, suggesting its ability to effectively escape from lysosomes (Figure S3). Together, these findings demonstrate that this delivery system possesses cross-cell-type universality, which is of great significance for future treatment of intracellular infections in different host cells.

### 2.4. Characterization of CRO Binding to ZF5.3-BSA and Drug Loading Quantification

**Figure 6:**
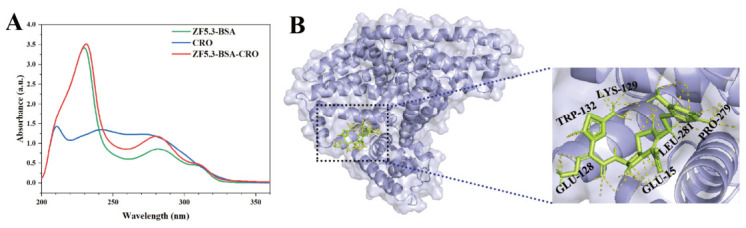
Binding of CRO to BSA. (**A**) UV-Vis absorption spectra of ZF5.3-BSA, CRO, and ZF5.3-BSA-CRO; (**B**) molecular docking simulation results of CRO and BSA.

Having confirmed the efficient cellular uptake of ZF5.3-BSA, we proceeded to evaluate its drug-loading capacity. Ceftriaxone (CRO), a third-generation cephalosporin antibiotic, was selected as the model antibiotic and incorporated into the delivery system by co-incubation with ZF5.3-BSA. UV-Vis absorption spectroscopy (Figure 6A) showed that CRO-loaded ZF5.3-BSA (ZF5.3-BSA-CRO) exhibited a significant increase in absorbance in the 250–290 nm region compared to ZF5.3-BSA alone, attributable to the superimposition of the CRO absorption spectrum. The binding ratio of CRO to BSA was calculated based on the changes in absorbance, yielding a CRO-to-BSA molar ratio of approximately 1.02 ± 0.05, indicating that each BSA molecule binds approximately one CRO molecule. To further confirm the binding ratio, the amount of free CRO was determined using an ultrafiltration-HPLC method. The results showed that the binding ratio of BSA to CRO was approximately 1:1 (Figure S4), which is consistent with the UV-Vis and molecular docking results. Molecular docking analysis was subsequently performed to elucidate the binding mode between BSA and CRO. As shown in Figure 6B, CRO was stably accommodated within the hydrophobic cavity of the BSA subdomain. The binding energy was −3.98 kcal/mol calculated by AutoDock Vina (v1.2.7), indicating spontaneous formation of a stable complex with favorable conformational complementarity. Detailed interaction analysis revealed that CRO engaged multiple key residues of BSA through a network of interactions: the side chain of CRO formed hydrogen bonds with residues TRP132, GLU128, GLU15, and LYS129, while the cephem core established extensive hydrophobic contacts with LEU281 and PRO279. These cooperative interactions drove stable encapsulation of CRO within the BSA cavity, further corroborating the 1:1 stoichiometric binding ratio at the molecular level. Furthermore, docking simulations revealed that cefixime and cephalexin could also bind to the hydrophobic cavity of BSA with a 1:1 stoichiometry, exhibiting binding energies of less than −3.5 kcal/mol, indicative of strong binding affinity (Figure S5). These results suggest that the delivery strategy constructed in this study has the potential to serve as a universal platform for intracellular antibiotic delivery. In vitro release experiments revealed that CRO exhibited a sustained release profile from the BSA-CRO complex, with a cumulative release rate of approximately 70% at 8 h (Figure S6), which is favorable for maintaining effective intracellular drug concentrations.

BSA bound to CRO at a 1:1 stoichiometric ratio, which is similar to previously reported binding of BSA to other β-lactam antibiotics, such as cefazolin and cefoxitin, although the binding constants differed slightly, likely due to specific hydrogen-bonding interactions involving the side chain of CRO. Molecular docking revealed that CRO binds to the Site I hydrophobic cavity of BSA, which is consistent with the classical binding site of BSA for various small-molecule drugs. Notably, we verified the binding ratio by HPLC, excluding the potential interference of protein absorption in UV-Vis spectroscopy and thereby rendering the data more reliable. Furthermore, cefixime and cephalexin also exhibited similar binding modes, suggesting that this delivery platform may be applicable to a variety of cephalosporin antibiotics and holds potential for universality.

### 2.5. Construction of the Intracellular Infection Model and Evaluation of Intracellular Antibacterial Activity

**Figure 7:**
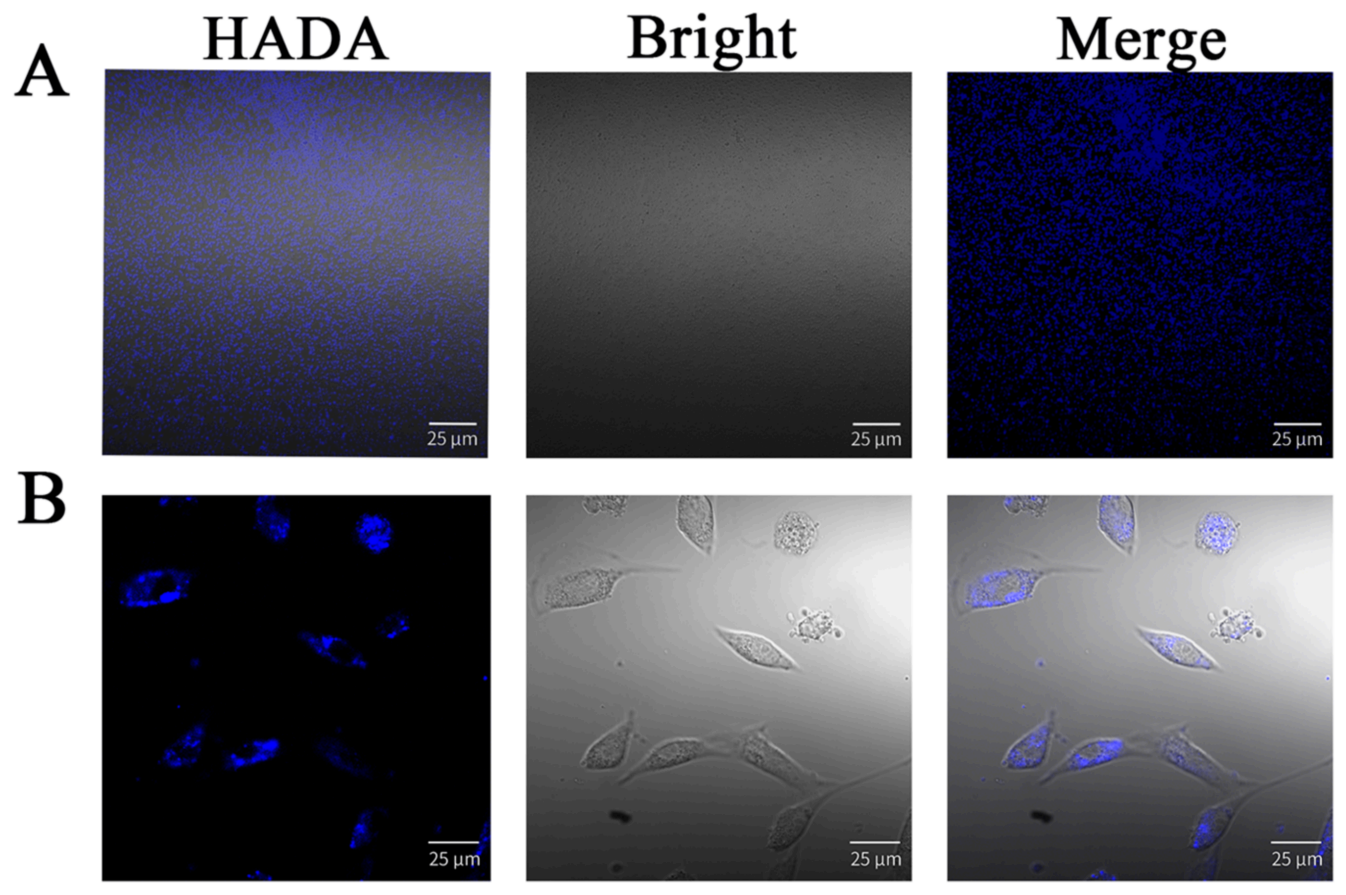
Validation of the intracellular *S. aureus* infection model. (**A**) Confocal microscopy image of *S. aureus* labeled with the HADA fluorescent probe, scale bar = 25 μm; (**B**) confocal microscopy image of 3T3 cells infected with HADA-labeled *S. aureus*, scale bar = 25 μm.

An intracellular infection model was established by infecting 3T3 cells with *Staphylococcus aureus* (*S. aureus*). To facilitate visualization of intracellular infection, *S. aureus* was first metabolically labeled using HCC-Amino-D-alanine hydrochloride (HADA) fluorescent probe. HADA-labeled bacteria exhibited bright blue fluorescence (Figure 7A). HADA-labeled *S. aureus* was then used to infect 3T3 cells, and the intracellular distribution of bacteria was examined by laser scanning confocal microscopy. As shown in Figure 7B, numerous blue fluorescence-labeled bacteria appeared as discrete puncta distributed within 3T3 cells, confirming successful invasion of *S. aureus* into host cells and validating the establishment of the intracellular infection.

**Figure 8:**
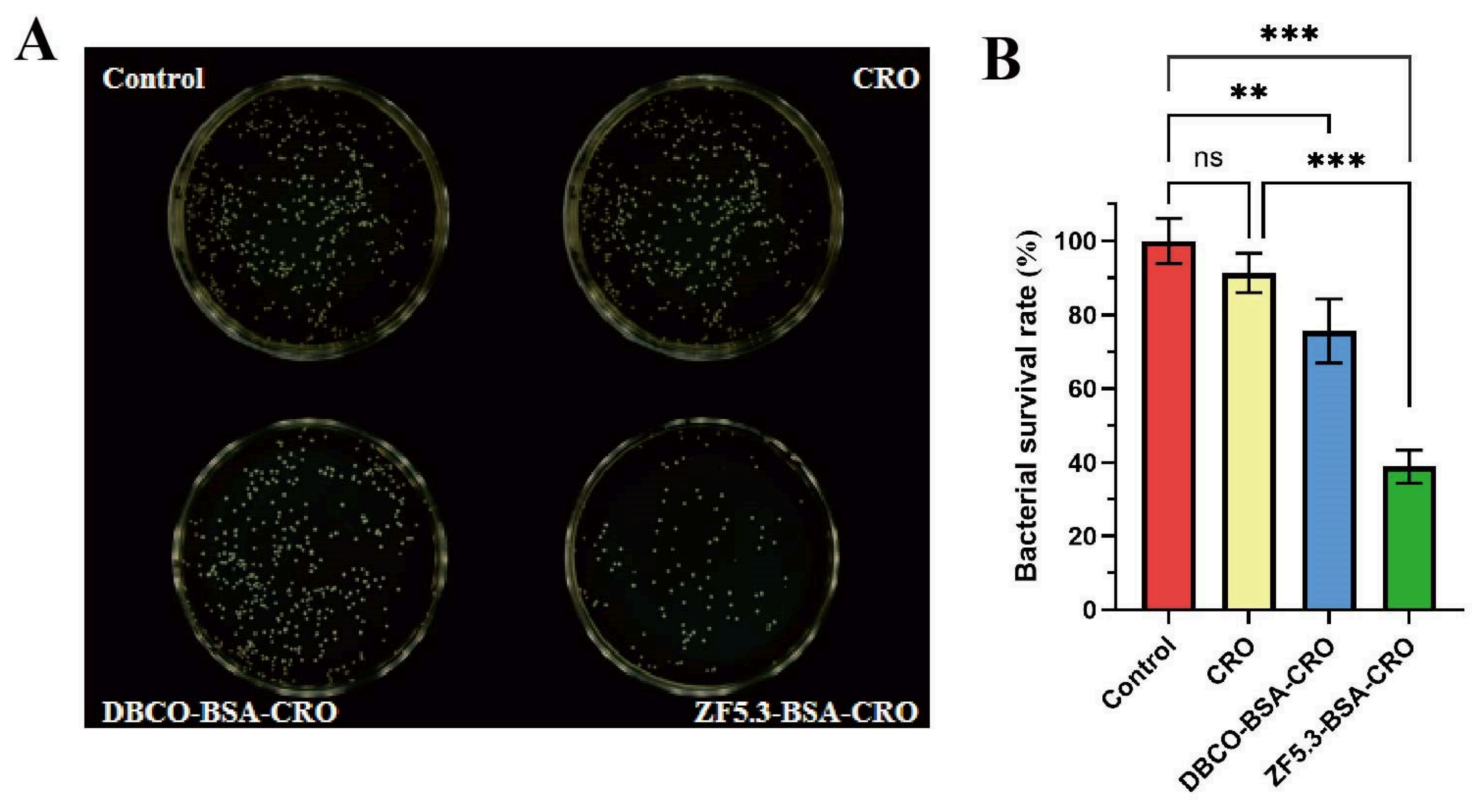
Evaluation of the antibacterial effect of ZF5.3-BSA-CRO. (**A**) Plate colony counts results of intracellular *S. aureus* in different treatment groups; (**B**) quantitative statistics of intracellular bacterial survival rates in each group. Data are presented as mean ± standard deviation (SD), n = 3 biological replicates. ns, no significant difference; ** *p* < 0.01; *** *p* < 0.001.

The intracellular antibacterial activity of different treatment groups was subsequently evaluated. Colony-forming unit (CFU) counting results (Figure 8A) showed abundant bacterial colonies on the plates of the untreated control group, reflecting a high intracellular bacterial burden. In the free CRO treatment group, colony counts were not significantly different from the control group, indicating that free CRO exerted negligible intracellular antibacterial activity. The DBCO-BSA-CRO group demonstrated slight antibacterial effects, while the ZF5.3-BSA-CRO group showed a marked reduction in colony counts, indicating substantially enhanced antibacterial activity. Quantitative analysis of bacterial survival rates (Figure 8B) further confirmed that the intracellular bacterial survival rate in the ZF5.3-BSA-CRO group was significantly lower than that in both the free CRO and DBCO-BSA-CRO groups. Notably, the antibacterial efficacy of DBCO-BSA-CRO exceeded that of free CRO, suggesting that DBCO-modified BSA itself can achieve a degree of intracellular delivery through endocytosis. The conjugation of ZF5.3 further markedly enhanced delivery efficiency, enabling more effective intracellular transport of CRO and resulting in superior intracellular antibacterial performance. Compared with free CRO, which was nearly ineffective, ZF5.3-BSA-CRO significantly reduced the survival rate of intracellular *S. aureus*. This result is consistent with the general trend reported for antibiotic delivery using cell-penetrating peptides or cationic polymers. However, the advantage of this study lies in the excellent biocompatibility and biodegradability of the BSA carrier itself, and the fact that ZF5.3 avoids the lysosomal trapping issue associated with conventional cell-penetrating peptides. Notably, DBCO-BSA-CRO also exhibited a certain antibacterial effect, suggesting that unmodified BSA can achieve a modest level of intracellular delivery via endocytosis, which is consistent with the partial internalization of BSA itself. Nevertheless, the introduction of ZF5.3 greatly enhanced the antibacterial efficacy, further confirming the critical role of lysosomal escape in intracellular drug activity. The biocompatibility of ZF5.3-BSA-CRO was verified by CCK-8 assay (Figure S7), which showed no obvious cytotoxicity in 3T3 cells.

## 3. Materials and Methods

### 3.1. Materials

BSA and Hoechst 33342 live-cell nuclear stain were purchased from Beyotime Biotechnology (Shanghai, China). DBCO-NHS ester was obtained from Adamas Reagent Co., Ltd. (Shanghai, China). Fluorescein isothiocyanate (FITC) was purchased from Aladdin Biochemical Technology Co., Ltd. (Shanghai, China). Ceftriaxone (CRO) was obtained from Rhawn Reagent Co., Ltd. (Shanghai, China). Sterile phosphate-buffered saline (PBS) was used as the working buffer throughout all experiments. All reagents were of analytical grade. The HeLa human cervical carcinoma cell line (STR authenticated) was purchased from Changsha Abiowell Biotechnology Co., Ltd. (Changsha, China, Cat# AW-CCH138). The 3T3-L1 mouse embryonic fibroblast cell line (STR authenticated), a clonal subline of Swiss albino 3T3 cells, was purchased from Wuhan Zishan Biotechnology Co., Ltd. (Wuhan, China, Cat# STCC20001P), with equivalent deposit as ATCC CL-173 (RRID: CVCL_0123).

### 3.2. Synthesis of ZF5.3 and Conjugation with BSA

ZF5.3 was synthesized by Fmoc solid-phase peptide synthesis (SPPS) and purified by high-performance liquid chromatography (HPLC). To facilitate bioorthogonal conjugation with BSA, an azide-modified lysine residue was incorporated at the C-terminus of ZF5.3 during solid-phase synthesis, yielding the sequence Ac-YSCNVCGKAFVLSRHLNRHLRVHRRATK(N_3_)-NH_2_. BSA was functionalized with DBCO groups by reaction with DBCO-NHS ester to generate DBCO-BSA. The resulting DBCO-BSA was subsequently conjugated with ZF5.3 via strain-promoted azide-alkyne cycloaddition (click chemistry) to obtain the ZF5.3-BSA conjugate.

Specifically, BSA was dissolved in PBS (pH 8.0) at a concentration of 10 mg/mL. DBCO-NHS ester was dissolved in DMSO to prepare a 5 mM stock solution. BSA and DBCO-NHS ester were mixed at a molar ratio of 1:3 and stirred at room temperature in the dark overnight to yield DBCO-BSA. The reaction mixture was purified by centrifugal ultrafiltration using a 10 kDa molecular weight cut-off (MWCO) membrane to remove unreacted small molecules. ZF5.3 was then added to DBCO-BSA at a molar ratio of ZF5.3 to DBCO-BSA of 1:1 and reacted at room temperature for 1 h to obtain the ZF5.3-BSA conjugate, which was stored at 4 °C until further use.

### 3.3. Preparation and Characterization of FITC-Labeled BSA Conjugates

To enable fluorescence-based tracking of intracellular BSA uptake, FITC-labeled BSA conjugates were prepared. DBCO-BSA was first prepared as described in Section 3.2. FITC was dissolved in DMSO to prepare a 5 mM stock solution and added to DBCO-BSA at a molar ratio of BSA to FITC of 1:3. The mixture was stirred at room temperature in the dark for 6 h. Unreacted FITC was removed by centrifugal ultrafiltration using a 10 kDa MWCO membrane to yield purified DBCO-BSA-FITC. ZF5.3 was then added to DBCO-BSA-FITC at a molar ratio of ZF5.3 to DBCO-BSA-FITC of 1:1 and reacted at room temperature for 1 h to form the ZF5.3-BSA-FITC conjugate. This fluorescently labeled conjugate was used in subsequent cellular uptake experiments to directly monitor the intracellular distribution of BSA. Fluorescence emission spectra were recorded using a microplate reader (Shanghai Flash Spectrum Biotechnology Co., Ltd., Shanghai, China) with excitation at 488 nm (slit width: 20 nm) and emission collected over 500–600 nm. Samples including DBCO-BSA-FITC (15 μM), DBCO-BSA (equivalent concentration), and free FITC (45 μM, as control) were diluted in PBS (pH 7.4) and measured in 150 μL volumes.

### 3.4. SDS-PAGE Electrophoresis

Protein samples were resolved by SDS-PAGE using a 12% separating gel and a 5% stacking gel. Electrophoresis was performed at 80 V during stacking and 120 V during separation. Following electrophoresis, gels were either imaged under fluorescence or stained with Coomassie Brilliant Blue for protein band visualization.

### 3.5. Cellular Uptake and Validation of ZF5.3-Mediated Intracellular Delivery

HeLa cells were seeded in confocal dishes (for confocal imaging) and 12-well plates (for flow cytometry) and cultured until reaching approximately 70% confluence. Cells were then incubated with DBCO-BSA-FITC or ZF5.3-BSA-FITC diluted in DMEM at a final concentration of 8 μmol/L (calculated on a protein basis) and incubated at 37 °C for 4 h. Following incubation, cells were washed three times with PBS and stained with Hoechst 33342 solution in the dark for 15 min, followed by two additional PBS washes. Cells in confocal dishes were directly imaged under a laser scanning confocal microscope to assess the intracellular distribution of FITC (green) and Hoechst (blue) fluorescence. Cells in 12-well plates were detached by trypsinization, collected by centrifugation, resuspended in PBS, and analyzed by flow cytometry for FITC channel fluorescence intensity. Untreated cells were used as a negative control. Mean fluorescence intensity (MFI) was calculated for each treatment group to evaluate the enhancement of intracellular BSA delivery by ZF5.3. (Each experiment contained three technical replicates and was repeated three independent biological times (n = 3).)

### 3.6. Preparation of CRO-Loaded ZF5.3-BSA Conjugate and Drug Loading Quantification

Ceftriaxone (CRO), a third-generation cephalosporin antibiotic, was selected as the model drug for constructing the drug-loaded delivery system. ZF5.3-BSA and CRO were mixed at a molar ratio of 1:3 and incubated with stirring at room temperature for 6 h to allow CRO to bind within the hydrophobic pocket of BSA. Following incubation, unbound free CRO was removed by centrifugal ultrafiltration using a 10 kDa MWCO membrane, and the ZF5.3-BSA-CRO conjugate was collected. To quantify drug loading, ZF5.3-BSA and ZF5.3-BSA-CRO samples at equivalent concentrations were diluted in PBS and absorbance was measured at 241 nm. ZF5.3-BSA served as the background reference to subtract the intrinsic protein absorption at 241 nm. The concentration of CRO in the conjugate was calculated based on a pre-established CRO standard calibration curve, from which the drug loading content was determined.

### 3.7. Molecular Docking Analysis of CRO with BSA

The crystal structure of BSA was downloaded from the RCSB Protein Data Bank (PDB ID: 8WDD, resolution 2.47 Å). Crystallographic water molecules, small-molecule ligands, and heteroatoms were removed using PyMOL (v3.1.6.1), and hydrogen atoms were added to the protein via AutoDockTools (v1.5.7). Based on the BSA structure (PDB ID: 8WDD), a docking grid box of 16 Å in size was centered on the hydrophobic cavity of BSA, and semi-flexible molecular docking was performed using AutoDock Vina (v1.2.7). After docking, the predicted binding affinity (in kcal/mol) was extracted, and the optimal binding conformation was determined. The non-covalent interactions between BSA and CRO, including hydrogen bonds, hydrophobic interactions, and van der Waals forces, were analyzed using PyMOL and Discovery Studio (v2025 Client).

### 3.8. Establishment of the Intracellular Bacterial Infection Model

An intracellular infection model was established using *Staphylococcus aureus* (ATCC 29213) and 3T3 cells. *S. aureus* was inoculated into LB broth and cultured at 37 °C with agitation at 220 r/min until reaching the logarithmic growth phase (OD_600_ = 0.6–0.8). The bacterial suspension was washed twice with PBS and resuspended in antibiotic-free DMEM for subsequent use. 3T3 cells were seeded in confocal dishes and maintained at 37 °C in a humidified atmosphere with 5% CO_2_ until reaching 70–80% confluence. The culture medium was removed, and bacterial suspension was added at a multiplicity of infection (MOI) of 50, followed by incubation at 37 °C for 1 h to allow cellular infection. Following infection, cells were washed three times with PBS to remove non-internalized extracellular bacteria, and subsequently incubated with complete medium containing 100 μg/mL gentamicin at 37 °C for 4 h to eradicate remaining extracellular bacteria. The gentamicin-containing medium was then removed, and cells were washed three times with PBS prior to further experiments [31,32].

For fluorescence imaging of intracellular infection, *S. aureus* was metabolically labeled with HADA to facilitate visualization of bacterial localization within host cells [33,34]. When the bacteria grew to the logarithmic phase, HADA was added to the bacterial culture at a final concentration of 1 mmol/L, and co-cultured at 37 °C with shaking at 220 r/min for 45 min. After metabolic fluorescent labeling, the treated bacteria were used to infect 3T3 cells.

### 3.9. Intracellular Antibacterial Assay

After the establishment of the intracellular infection model, cells were treated with PBS (negative control), free CRO, DBCO-BSA-CRO, or ZF5.3-BSA-CRO, with a final CRO concentration of 10 μM in all antibiotic-containing groups. Cells were incubated at 37 °C for 6 h. After incubation, the supernatant was discarded and the cells were washed three times with PBS. Cells were then lysed by adding 200 μL of 0.5% Triton X-100 lysis buffer (in PBS) per well and incubating on ice for 15 min with repeated pipetting to ensure complete lysis. The cell lysates were serially diluted, and equal volumes of each dilution were plated onto LB agar plates. Plates were incubated at 37 °C for 12–16 h. Colonies were counted on plates containing 100–500 colonies. Since identical dilution factors and plating volumes were adopted across all groups, dilution constants offset each other during calculation. The bacterial survival rate was calculated according to the following formula:

$$Bacterial\;survival\;rate\;(\%)=\frac{Mean\;colony\;count\;of\;treatment\;group}{Mean\;colony\;count\;of\;PBS\;control\;group}\times 100\%$$

(Each experiment contained three technical replicates and was repeated three independent biological times (n = 3).)

### 3.10. Instrumentation

The main instruments used in this study are listed as follows: High-performance liquid chromatography (HPLC): P3140 (Elite, Dalian, China); Gel imaging system: ChemiScope 6200 (Qinxiang, Shanghai, China); Microplate reader: SuperMax 3200 (Shanpu, Shanghai, China); Laser scanning confocal microscope: NCF950 (Nexcope, Ningbo, China); Flow cytometer: LongCyte C3140 (Longsee Biotech, Beijing, China).

## 4. Conclusions

In this study, we successfully constructed a ZF5.3-modified BSA-based intracellular delivery system. ZF5.3 was stably and efficiently conjugated to BSA through DBCO-mediated click chemistry. Comprehensive characterization and cellular experiments confirmed that ZF5.3 significantly enhances the cellular internalization and intracellular transport of BSA, with flow cytometric quantification demonstrating a more than 5-fold improvement in delivery efficiency. Fluorescence co-localization analysis revealed that ZF5.3-mediated cargo is mainly distributed in the cytoplasm and does not completely co-localize with lysosomal markers, suggesting its ability to effectively escape from lysosomes. UV-Vis spectroscopy, HPLC, and molecular docking simulations revealed that CRO binds stably within the Site I hydrophobic cavity of BSA at a 1:1 stoichiometric ratio without disrupting the native BSA conformation. In vitro release profile of CRO from the BSA-CRO complex in PBS. Cumulative release reached approximately 70% at 8 h and then plateaued, indicating sustained release behavior. In vitro intracellular antibacterial assays demonstrated that ZF5.3-BSA-CRO exerted significantly enhanced therapeutic efficacy against intracellular *S. aureus* infection compared to all control groups. The delivery system is straightforward to construct, exhibits excellent biocompatibility, and represents a functional upgrade of the classical BSA carrier platform. Overall, this work provides a highly promising delivery strategy for the treatment of intracellular bacterial infections and intracellular-targeted therapy, with broad potential for clinical translation.

## Data Availability

The data presented in this study are available on request from the corresponding authors.

## Supplementary Materials

The following supporting information can be downloaded at: https://www.mdpi.com/article/10.3390/molecules31132215/s1, Figure S1. Native-PAGE analysis of BSA-CRO and ZF5.3-BSA-CRO complexes with silver staining. Figure S2. Laser confocal microscopy imaging of 3T3 cells in different treatment groups. Figure S3. Lysosomal escape assay. Figure S4. HPLC analysis of CRO binding to BSA. Figure S5. Binding of antibiotics to BSA. Figure S6. In vitro release profile of CRO from the ZF5.3-BSA-CRO complex in PBS (pH 7.4, 37 °C). Figure S7. Cell viability of 3T3 cells after 24 h treatment with ZF5.3, ZF5.3-BSA, and ZF5.3-BSA-CRO, as determined by CCK-8 assay.

## Abbreviations

The following abbreviations are used in this manuscript:

BSA	Bovine Serum Albumin
CRO	Ceftriaxone
SPPS	Solid-Phase Peptide Synthesis
UV-Vis	Ultraviolet-Visible Spectroscopy
HPLC	High-Performance Liquid Chromatography
LC-MS	Liquid Chromatography-Mass Spectrometry
FITC	Fluorescein Isothiocyanate
MFI	Mean Fluorescence Intensity
HADA	HCC-Amino-D-alanine hydrochloride
MOI	Multiplicity of Infection
CFU	Colony-Forming Units
CCK-8	Cell Counting Kit-8
