# Supplementary material for: The Fabrication of Protein Carriers for Intracellular Delivery of Antibiotics Against Intracellular Bacterial Infection
Source: Molecules. 2026 Jun 24;31(13):2215. doi: 10.3390/molecules31132215 (PMC13363164; doi:10.3390/molecules31132215)
Supplement: Supplementary file 1 [file molecules-31-02215-s001.zip › molecules-4350863-supplementary.pdf]

Supporting Information

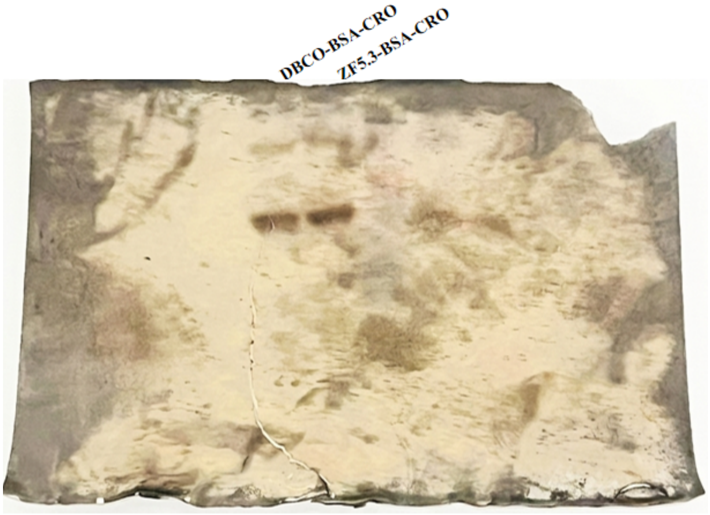

Figure S1. Native-PAGE analysis of BSA-CRO and ZF5.3-BSA-CRO complexes with silver staining. Both complexes migrated as single, sharp bands without smearing, indicating homogeneity and integrity.

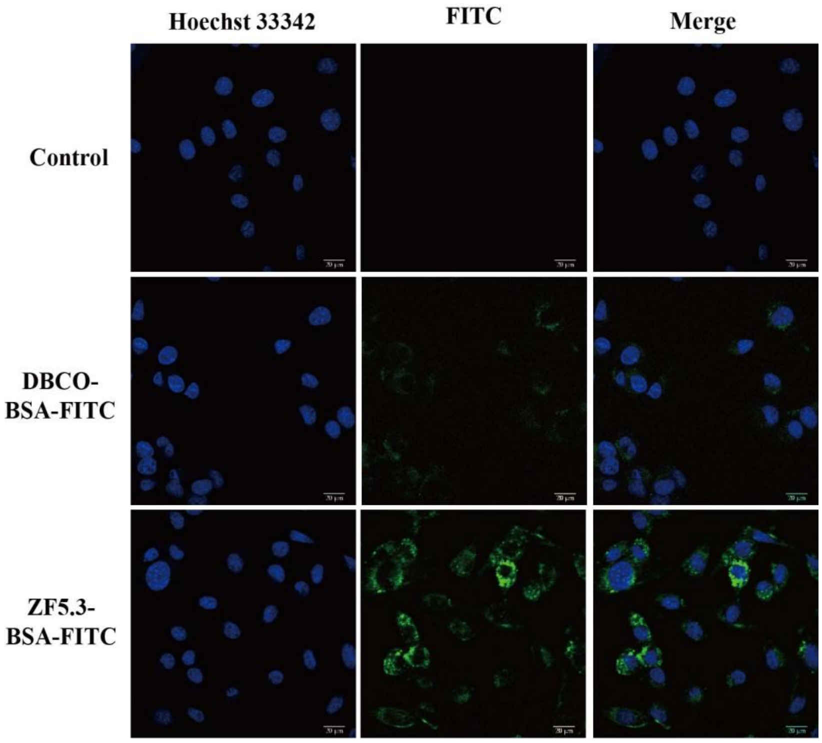

Figure S2. Laser confocal microscopy imaging of 3T3 cells in different treatment groups (blue: Hoechst 33342 nuclear staining; green: FITC-labeled protein; Scale bar = 20 μm)

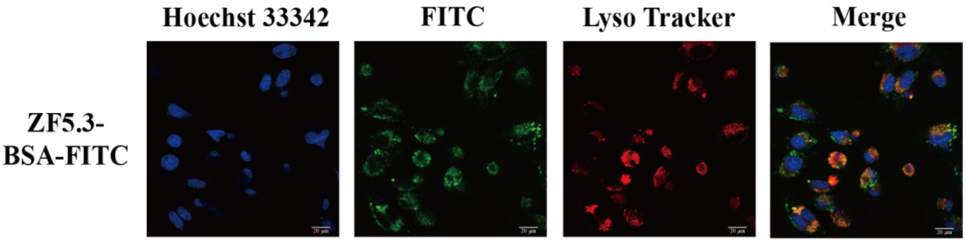

Figure S3. Lysosomal escape assay. 3T3 cells treated with ZF5.3-BSA-FITC showing diffuse cytoplasmic green fluorescence and incomplete colocalization with lysosomal marker. Scale bar = 20 μm.

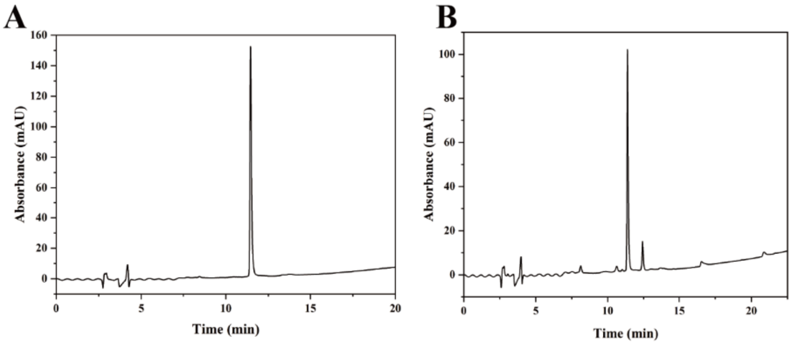

Figure S4. HPLC analysis of CRO binding to BSA. (A) Chromatogram of free CRO standard (3-fold molar excess relative to BSA); (B) Chromatogram of the ultrafiltration flow-through fraction of the BSA-CRO mixture. The reduction in peak area in (B) indicates that a portion of CRO was bound to BSA. Based on peak area integration, the binding ratio of CRO to BSA was calculated to be approximately 1:1.

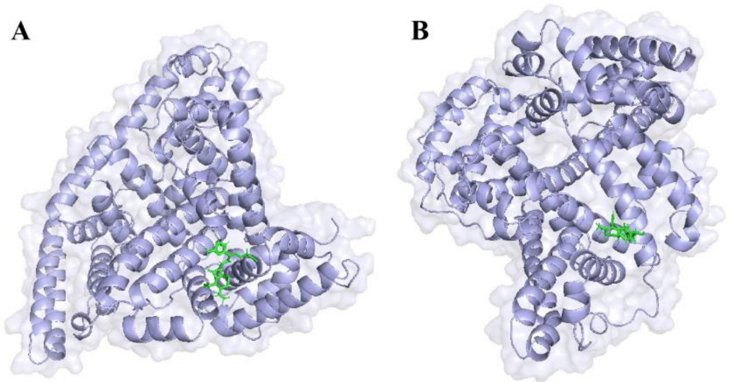

Figure S5. Binding of antibiotics to BSA. (A) Molecular docking simulation results of cefixime and BSA; (B) Molecular docking simulation results of cephalexin and BSA.

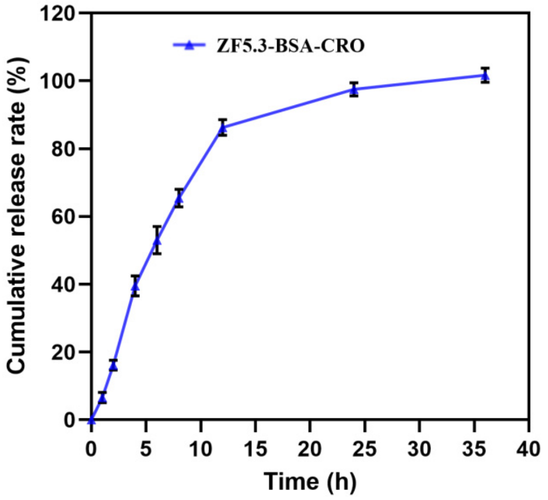

Figure S6. In vitro release profile of CRO from the ZF5.3-BSA-CRO complex in PBS (pH 7.4, 37 °C). Cumulative release reached approximately 70% at 8 h and then plateaued, indicating sustained release behavior.

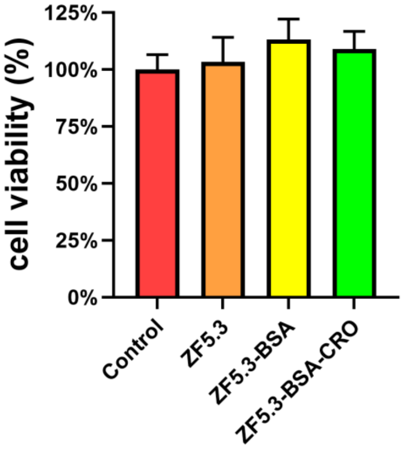

Figure S7. Cell viability of 3T3 cells after 24 h treatment with ZF5.3, ZF5.3-BSA, and ZF5.3-BSA-CRO, as determined by CCK-8 assay.
